# Intestinal parasitic infections and their association with bruxism, tooth wear, and temporomandibular disorders in children in rural Egypt: a cross-sectional study

**DOI:** 10.1186/s12903-026-08751-3

**Published:** 2026-06-04

**Authors:** Radwa Rabie, Mona ELkashlan, Susan Saleh

**Affiliations:** 1 Department of Pediatric Dentistry and Dental Public Health, Faculty of Dentistry, Alexandria University, Alexandria, Egypt; 2 Faculty of Dentistry, Alexandria University, Champollion St., Alexandria, Azarita Egypt

**Keywords:** Intestinal parasitic infections (IPIs), Bruxism, TMD, Wear index, Children, Alexandria, Egypt

## Abstract

**Background:**

Bruxism is a common oral habit characterized by involuntary grinding of teeth, traditionally linked to multiple factors, including intestinal parasitic infections (IPIs). This study investigated the association between bruxism, as measured by tooth wear and temporomandibular disorders (TMDs), and the presence of IPIs in children.

**Methods:**

A comparative cross-sectional study was conducted at a governmental primary health unit in Alexandria, Egypt, between August 2023 to 2024. A convenience sample of 450 healthy children (aged 3–10 years), with Angle’s Class I occlusion, were recruited and their infection status was confirmed based on their stool analysis results (positive for IPIs, *n* = 225; negative for IPIs, *n* = 225). Parents completed a questionnaire on demographics and bruxism risk indicators. Bruxism was evaluated using parent reports, the Helkimo’s clinical Dysfunction Index for TMDs and masticatory muscle assessment, and the Hugoson’s Tooth Wear Index for dental wear. Descriptive statistics and multivariable logistic regression models were used for data analysis.

**Results:**

Of the 450 children, 50.6% were females. The mean age was significantly higher (7.38 ± 1.94) in the positive group than in the negative group (6.71 ± 1.52). Reported bruxism was significantly more frequent in participants with IPIs (61.5%, *p* = 0.001). Mild TMD was significantly more prevalent in children with IPIs (+ ve = 60.1%, *p* = 0.001). The mean number of affected teeth with wear was (2.72 ± 3.29) in the positive group and (2.50 ± 3.41) in the negative group. IPIs were significantly associated with bruxism (AOR = 2.08; 95% CI: 1.32–3.29; tooth wear (AOR = 1.64; 95% CI: 1.08–2.47), and TMD (AOR = 1.85; 95% CI: 1 0.23–2.80).

**Conclusion:**

Children with IPIs showed a higher occurrence of bruxism as reported by their mothers, which was associated with mild temporomandibular disorders (TMDs) and increased tooth wear.

**Supplementary Information:**

The online version contains supplementary material available at 10.1186/s12903-026-08751-3.

## Introduction

Oral health is an integral part of general health and overall well-being. Bruxism is an involuntary parafunctional habit characterized by involuntary jaw muscle activity causing teeth to grind or clench, often occurring without conscious awareness [1, 2]. Bruxism is especially prevalent among children, and it tends to diminish with age [2, 3]. Research reveals that the prevalence of bruxism affects children at rates ranging from 3.5% to 40.6% with no gender predilection [1, 4]. Its etiology is multifactorial, encompassing respiratory problems, sleep disturbances, psychosocial stressors, and gastrointestinal conditions, including intestinal parasitic infections. In addition, it leads to multiple adverse outcomes affecting the stomatognathic system, including dental wear, masticatory muscle discomfort and pain, headache and temporomandibular disorders (TMDs) [5, 6].

Recent international consensus led by Manfredini et al. has redefined bruxism as a masticatory muscle activity characterized by repetitive or sustained tooth grinding, clenching, or bracing of the jaw muscles, occurring during sleep or wakefulness, rather than strictly a disorder [7]. This “new vision,” formalized through tools like the Standardized Tool for the Assessment of Bruxism (STAB), employs a multidimensional continuum approach [8]. Axis A evaluating status and consequences via self-reports, clinical exams (e.g., tooth wear, TMD signs), while Axis B addresses risk factors like psychosocial stressors, sleep issues, and gastrointestinal conditions such as IPIs [8].While the STAB framework supports a nuanced pediatric diagnosis [9], A critical gap still remains in large-scale epidemiological studies that apply this multidimensional approach to evaluate the functional consequences of bruxism in relation to systemic risk indicators like IPIs.

Intestinal parasitic infections (IPIs) are among the most widespread diseases globally [10]. According to the World Health Organization (WHO, 2023) estimates, IPIs affect approximately 1.5 billion individuals, which corresponds to about 24% of the world’s population [11]. Approximately 600 million school-aged children worldwide have experienced at least one episode of intestinal parasitic infection [12]. In Egypt, the prevalence among school children has been reported to be 56.3% [13]. IPIs disproportionately burden low socioeconomic groups due to inadequate sanitation, limited access to clean water, overcrowding, poor hygiene practices, and contaminated food/soil exposure, hallmarks of rural settings. In Egypt, rural residence adds 40% elevated odds of infection via soil-transmitted helminths. Despite the high endemicity in rural Egypt, the specific impact of these infections on the orofacial health and masticatory function of Egyptian children remains under documented [14–16].

These infections represent a significant public health problem associated with iron deficiency anemia, stunted growth, gastrointestinal disturbances, headaches, sleep disruptions, perianal itching, and bruxism [10, 17, 18]. Several studies have indicated a potential association between bruxism and intestinal parasitic infections [17, 19, 20]. However, the existing evidence is often conflicting; while some research suggests a significant correlation [19, 21, 22], other studies found no significant link [23], often due to small sample sizes or a reliance on subjective parental reports. Furthermore, the potential for IPIs to serve as a risk indicator for more severe clinical manifestations, such as Temporomandibular Disorders (TMDs), has not been adequately addressed in the pediatric literature.

Since understanding the factors associated with bruxism is essential for its treatment, prevention and quality of life [24], there is a a need for research that transcends perception-based reporting in favor of standardized, clinically-validated diagnostic protocols. This study aimed to explore the association between stool-confirmed intestinal parasitic infections and bruxism, tooth wear and TMD in a group of Egyptian children. The null hypothesis stated that there is no significant association between intestinal parasitic infections and these outcomes.

## Methods

This comparative cross-sectional study was carried out at a primary health unit affiliated with the Egyptian Ministry of Health in Alexandria, based in the North Delta region of Egypt from August 2023 to August 2024. The study was conducted after receiving the approval of the research ethics committee, Faculty of Dentistry, Alexandria University with the number: (IRB 00010556 – IORG 0008839 - #042742022), and the approval of the Ministry of Health. Informed consents were obtained from the caregivers to participate in the study after explaining the study’s objectives and assuring them of the absolute confidentiality of the collected data.

Participants were included if they were aged 3 to 10 years old with Angle class I occlusion, and children with chronic systemic diseases who took drugs that may affect the central nervous system or children with serious psychiatric or neurological disorders and those who were treated with anthelminthic drugs in the last two months before recruitment were excluded.

The sample size was estimated assuming 80% study power and 5% alpha error according to a study that reported that children with gastrointestinal disorders had a significantly higher (2.090-fold) probability of having bruxism [25]. The prevalence of bruxism among healthy children was 15.45%. Based on the previous assumptions using binomial logistic regression, a total sample of 361 children was calculated, increased to 433 ≈ 450 children to make up for the 20% non-response rate. Total sample size = Number per group x Number of groups = 225 × 2 = 450 children. The sample size was based on Rosner’s method [26] calculated by G*power 3.1.9.7 [27].

Participants were recruited and their infection status was confirmed by the health unit parasitological laboratory. Based on their stool analysis, children were categorized into two groups: the infected positive group (*n* = 225), comprising those with positive stool analysis results and the comparative negative group (*n* = 225), comprising those with negative findings.

Data were collected using a structured examination questionnaire comprising two main sections. [Additional File 1] The first section involved a structured interview with each child’s caregiver. This section captured information on the child’s demographic profile (age in years and sex), parental education level, reported bruxism, and bruxism-related history. Parents were asked about the frequency of bruxism episodes over the past three months and whether bruxism had occurred on a daily basis. Additional questions addressed oral habits associated with bruxism [28, 29], sleep quality, and potential risk indicators, including recent stressful life events [30] and exposure to violence [31–33]. Responses were recorded using a standardized format with the following options: “Yes,” “No,” or “I don’t know.” The questionnaire was evaluated for content and face validity. Content validity was assessed by a panel of nine experts in dental public health and pediatric dentistry, resulting in average Item‑Content Validity Index (I‑CVI) values of 0.92, indicating excellent agreement on item relevance [34]. Face validity was confirmed by a small group of parents, ensuring clarity and appropriateness of all items. Minor adjustments were made based on their feedback before final administration.

The clinical examination included an extraoral evaluation of the temporomandibular joint (TMJ) and muscles of mastication using the Helkimo Dysfunction Index [35] to detect any signs of functional impairment. The examination included mandibular opening, observation of any deviation during opening, TMJ dysfunction, TMJ pain and the preauricular region, and palpation of the masticatory muscles for pain. The scores assigned to the five symptoms were summed up. Each individual gained a score ranging from 0 to 5 for each symptom according to the degree of impairment; 0 = no impairment, or no pain; 1 = mild impairment or palpable pain; 5 = severe impairment or severe pain with a palpebral reflex. An overall dysfunction score ranging from 0_25 points was subsequently calculated, where a higher score signifies a more acute disorder.

An intraoral examination included an assessment of each child’s teeth for the presence of wear facets, following the criteria of the Hugoson Wear Index [36]. All erupted teeth were assessed and assigned a score according to each degree of incisal or occlusal wear. The scoring criteria were as follows: Score of 0 indicated no wear or negligible wear of enamel, Score of 1 represented noticeable enamel wear or enamel worn through to dentin in single spots, Score of 2 corresponded to dentin wear up to one-third of the crown height, and a Score of 3 denoted dentin wear exceeding one third of the crown height. All examinations were conducted under natural daylight, with additional illumination provided by a portable light source to enhance visibility and diagnostic accuracy. Children were seated on a straight-backed chair, and standard disposable dental instruments (mirrors and probes) were used for inspection and palpation [18].

Data collection was completed in a single comprehensive session, including parental interview, dental examination, and stool analysis, to minimize non-response and incomplete data and a single calibrated dentist performed all assessments to ensure consistency. The intraexaminer agreement in terms of tooth wear and TMD was assessed by re-examining 20 children after one week and, and the Kappa values were 0.805 and 0.836 which indicated a substantial agreement.

### Statistical analysis

The dependent variable caregiver reported bruxism, tooth wear as assessed by the occlusal wear index and TMD as evaluated by TMJ dysfunction index. The independent variable **“**Intestinal parasitic infections” was assessed by stool analysis testing. Violent exposure, quality of sleep, adverse oral habits related to tooth wear and stressful life events were assessed by the caregiver’s interview. The confounders were age, sex, socioeconomic status, and parental educational level. The normality of quantitative variables (age, tooth wear scores, and TMD scores) was assessed using the Kolmogorov–Smirnov test and Q–Q plots. Age followed a normal distribution and was compared between groups using the independent t-test. In contrast, tooth wear and TMD scores were not normally distributed and were analyzed using the Mann–Whitney U test. Categorical variables were summarized as frequencies and percentages and analyzed using Pearson’s Chi-square test or Fisher’s Exact test, as appropriate. Binary logistic regression was performed to examine the association between independent variables and clinical outcomes (reported bruxism, tooth wear and TMD). Both unadjusted and adjusted odds ratios (UORs and AORs) were reported, along with 95% confidence intervals (CIs) and p-values. A p-value < 0.05 was considered statistically significant. All analyses were conducted using IBM SPSS Statistics for Windows, Version 26.0 (Armonk, NY, USA).

## Results

Among the study subjects, the mean age was significantly higher (7.38 ± 1.94 years) in the positive infected group compared to negative group (6.71 ± 1.52 years). Girls represented 59.1% of the positive infected group. Mothers with education levels less than secondary school represented 81.3% of the positive infected group. Fathers with education levels higher than secondary school represented 32.0% of positively infected children. [Table 1]

**Table 1:** Socio-demographic characteristics of the study subjects

		Intestinal Parasitic infections		*p*-value
		Positive(+ ve) (*n* = 225)	Negative(-ve) (*n* = 225)	
Age in years	Mean ± SD	7.38 ± 1.94	6.71 ± 1.52	< 0.001*
Sex: n (%)	Males	92 (40.9%)	130(57.8%)	< 0.001*
	Females	133(59.1%)	95(42.2%)	
Father’s education: n (%)	Illiterate	0 (0%)	0 (0%)	0.684
	Less than secondary	153(68.0%)	157(69.8%)	
	Secondary and higher	72(32.0%)	68(30.2%)	
Mother’s education: n (%)	Illiterate	18(8.0%)	34(15.1%)	< 0.001*
	Less than secondary	183(81.3%)	185(82.2%)	
	Secondary and higher	24(10.7%)	6(2.7%)	

*Statistically significant difference at *p* value < 0.05

Experiencing stressful life events was significantly associated with a positive stool analysis, with 36.5% of affected individuals reporting such events compared to 63.5% of those without (*p* = 0.001). Additionally, exposure to violence was strongly linked to a positive stool analysis (31.9%) versus a negative one (68.1%, *p* < 0.001). However, no significant differences were found regarding low quality of sleep (*p* = 0.241) or adverse oral habits (*p* = 0.828). [Table 2]

**Table 2:** Risk indicators related to bruxism among the study subjects

			Intestinal Parasitic infections		*p*-value
			Positive(+ ve) (*n* = 225)	Negative(-ve) (*n* = 225)	
Other oral habits	Bite down on hard objects	Yes	15 (38.5%)	24 (61.5%)	0.132
		No	210 (51.1%)	210 (48.9%)	
	Crushing hard candies	Yes	49 (46.7%)	56 (53.3%)	0.435
		No	176 (51.0%)	169 (49.0%)	
	Opening bottles with teeth	Yes	7 (100%)	0 (0.0%)	0.008*
		No	218 (49.2%)	225 (50.8%)	
	Adverse oral habits	Yes	58 (50.9%)	56 (49.1%)	0.828
		No	167 (49.7%)	169 (50.3%)	
Quality of sleep	Sleep disorders (sleep apnea)	Yes	6 (54.5%)	5 (45.5%)	0.760
		No	219 (49.9%)	220 (50.1%)	
	Snores or nightmares	Yes	3 (25.0%)	9 (75.0%)	0.079
		No	222 (50.7%)	216 (49.3%)	
	Low quality of sleep	Yes	7 (36.8%)	12 (63.2%)	0.241
		No	218 (50.6%)	213 (49.4%)	
Stressful life events	Birth of sibling	Yes	37 (39.4%)	57 (60.6%)	< 0.020*
		No	188 (52.8%)	168 (47.2%)	
	Change of address	Yes	1 (12.5%)	7 (87.5%)	0.032*
		No	224 (50.7%)	218 (49.3%)	
	Divorce of parents	Yes	0 (0.0%)	0 (0.0%)	--
		No	225 (50.0%)	225 (50.0%)	
	Death of family member	Yes	4 (26.7%)	11 (73.3%)	0.066
		No	221 (50.8%)	214 (49.2%)	
	Facing stressful life events	Yes	42 (36.5%)	73 (63.5%)	0.001*
		No	183 (54.6%)	152 (45.4%)	
Violent exposure	Physical or emotional abuse	Yes	43 (30.5%)	98 (69.5%)	< 0.001*
		No	182 (58.9%)	127 (41.1%)	
	Violent video games	Yes	7 (70.0%)	3 (30.0%)	0.201
		No	218 (49.5%)	222 (50.5%)	
	Overall violent exposure	Yes	46 (31.9%)	98 (68.1%)	< 0.001*
		No	179 (58.5%)	127 (41.5%)	

*Statistically significant difference at p value < 0.05

Bruxism was significantly more frequent in participants with a positive stool analysis (61.5%) compared to those with a negative result (38.5%, *p* = 0.001). The Helkimo index was also significantly higher in the positive group (1.04 ± 1.06 vs. 0.70 ± 1.00, *p* < 0.001), indicating greater TMD severity. TMD symptoms were present in 44% of participants, with a higher prevalence in the positive group (60.1% vs. 39.9%, *p* < 0.001). No significant difference was found in the number of affected teeth (2.72 ± 3.29 vs. 2.50 ± 3.41, *p* = 0.362). Tooth wear was more common in the positive group (54.4% vs. 45.6%), but this difference was not statistically significant (*p* = 0.105). [Table 3]

**Table 3:** Reported bruxism and Clinical examination of TMD and tooth wear among the study subjects

				Intestinal Parasitic infections		*p*-value	Overall
				Positive(+ ve) (*n* = 225)	Negative(-ve) (*n* = 225)		
Reported bruxism	Grinding or clenching while asleep/ daytime		Yes	88 (61.5%)	55 (38.5%)	0.001*	143 (31.8%)
			No	137(44.6%)	170 (55.4%)		307(68.2%)
Helkimo index score		Mean ± SD		1.04 ± 1.06	0.70 ± 1.00	< 0.001*	0.87 ± 1.04
		Median		1.00	0.00		0.00
		Min - Max		0–4	0–3		0.0–4.00
TMD symptoms: n (%)		Yes		119 (60.1%)	79 (39.9%)	< 0.001*	198 (44%)
		No		106 (42.1%)	146 (57.9%)		252 (56%)
Total affected teeth		Mean ± SD		2.72 ± 3.29	2.50 ± 3.41	0.362	2.61 ± 3.35
		Median		0.00	0.00		0.00
		Min - Max		0–15	0–12		0–15
Occurrence of tooth wear: n (%)		Yes		105 (54.4%)	88 (45.6%)	0.105	193 (42.9%)
		No		120 (46.7%)	137 (53.3%)		257(57.1%)

*Statistically significant difference at *p* value < 0.05

Binary logistic regression models were performed to identify factors associated with bruxism, tooth wear, and TMD occurrence. *For Bruxism*, Older age was significantly associated with increased odds of bruxism (AOR = 1.23; 95% CI: 1.08–1.39; *p* = 0.002). Children whose mothers had less than secondary education had higher odds of bruxism compared with those of illiterate mothers (AOR = 3.01; 95% CI: 1.36–6.64; *p* = 0.006). Positive intestinal parasitic infection (AOR = 2.08; 95% CI: 1.32–3.29; *p* = 0.002) and exposure to violence (AOR = 2.53; 95% CI: 1.52–4.22; *p* < 0.001) were significantly associated with brusxism. *For Tooth Wear*, Higher maternal education (secondary or above) was associated with reduced odds of tooth wear (AOR = 0.24; 95% CI: 0.07–0.76; *p* = 0.016). While the bivariate analysis revealed no statistically significant difference in tooth wear between children with and without IPI, a positive infection status was associated with a higher likelihood of tooth wear in the multivariable regression model, following adjustment for potential confounding variables. (AOR = 1.64; 95% CI: 1.08–2.47; *p* = 0.019). Other variables were not statistically significant. *For TMD*, Older age was associated with increased odds of TMD (AOR = 1.23; 95% CI: 1.09–1.38; *p* = 0.001). Males had lower odds of TMD compared with females (AOR = 0.59; 95% CI: 0.39–0.90; *p* = 0.013). Positive intestinal parasitic infection was significantly associated with TMD. (AOR = 1.85; 95% CI: 1.23–2.80; *p* = 0.003). [Table 4]

**Table 4:** Binary logistic regression models assessing the effect of risk indicators on bruxism, Tooth wear and TMD occurrence

			Bruxism						Tooth Wear						TMD				
			AOR		95% CI		*P* value		AOR		95% CI		*P* value		AOR		95% CI		*P* value
Age in years			1.23		1.08, 1.39		0.002*		0.95		0.85, 1.07		0.387		1.23		1.09, 1.38		0.001*
Sex	Males vs. Females	0.66		0.42, 1.03		0.068		1.40		0.93, 2.11		0.108		0.59		0.39, 0.90		0.013*	
Mother’s Education	Secondary and higher vs. Illiterate	2.30		0.69, 7.75		0.177		0.24		0.07, 0.76		0.016*		0.68		0.25, 1.87		0.458	
	Less than secondary vs. Illiterate	3.01		1.36, 6.64		0.006*		1.29		0.69, 2.39		0.427		0.83		0.44, 1.54		0.552	
Intestinal parasitic infections	Positive vs. Negative	2.08		1.32, 3.29		0.002*		1.64		1.08, 2.47		0.019*		1.85		1.23, 2.80		0.003*	
Low quality of sleep	Yes vs. No	2.03		0.76, 5.44		0.161		1.25		0.48, 3.25		0.650		1.06		0.39, 2.87		0.916	
Adverse oral habits related to tooth wear	Yes vs. No	0.76		0.47, 1.25		0.284		1.17		0.76, 1.82		0.475		1.00		0.64, 1.57		0.993	
Facing Stressful life events	Yes vs. No	1.24		0.76, 2.02		0.391		0.89		0.57, 1.40		0.608		0.92		0.58, 1.45		0.717	
Violent e; xposure	Yes vs. No	2.53		1.52, 4.22		< 0.001*		0.99		0.62, 1.56		0.948		1.45		0.84, 2.18		0.208	

*Statistically significant difference at *p* value < 0.05, AOR: Adjusted Odds Ratio, CI: Confidence Interval

## Discussion

This comparative cross-sectional study investigated the association between intestinal parasitic infections (IPIs) and bruxism, tooth wear and TMD in a sample of 450 Egyptian children aged 3–10 years. The study revealed that IPIs might be a significant indicator of bruxism, TMD symptoms and tooth wear. Age increased the likelihood of bruxism and TMD symptoms. Males had lower odds of developing TMD compared to females. Higher maternal education was associated with significantly lower odds of tooth wear. Other factors such as adverse oral habits, stressful life events, poor sleep quality, and violent exposure showed no significant association with either TMD or tooth wear. These findings suggest a potential association between IPIs and bruxism, TMD and tooth wear, therefore, the null hypothesis is rejected.

The results showed that intestinal parasitic infections (IPIs) were detected in older children, which is consistent with the findings of Yazgan et al. [37] who reported that the prevalence of intestinal parasitic infections increased with age, possibly because older children spend more time outdoors, often playing with soil and dirt especially in rural areas with less supervision and personal hygiene, thereby increasing the risk of infection. It was also reported that females experienced more IPIs than males, which was in line with some previous studies [38, 39]. This may be attributed to girls may assist more with domestic chores involving water, food preparation, or caring for younger siblings, as well as possible biological differences in the immune response between males and females. It was also reported that positive stool analysis was significantly lower in children whose parents had higher educational levels, which was consistent with previous studies [37, 40] likely because of better sanitation and hygiene practices, as well as greater awareness about the risk of parasitic infection and self-cleaning. The present study also revealed that increasing age increased the odds of having bruxism, which is consistent with the findings of other study [41], which suggested that bruxism tends to increase with age, which may be attributed to increased exposure to recurrent IPIs. It was also reported that higher mother’s education decreases the odds of bruxism, which is consistent with previous studies [42, 43] because awareness and education may therefore play a role in reporting bruxism and seeking medical consultation. The present study also showed that more than half of the children whose mothers reported bruxism were among those with a positive stool analysis. This finding was consistent with those of previous studies [17, 19, 20, 22, 37, 40] which suggested that intestinal parasitic infections could play a role in bruxism etiology. One possible explanation is that intestinal parasites produce metabolites known as nonspecific proteins, which are toxic substances secreted throughout their life cycles. These toxic compounds may potentially trigger the brain to induce teeth grinding due to the close communication between the gut and brain via the vagus nerve [20]. Beyond toxic metabolites, IPIs may further trigger muscle hyperactivity by inducing systemic inflammation and micronutrient deficiencies, specifically in Magnesium and Vitamin B12, which are established risk factors for neuromuscular instability [44]. Additionally, it has been reported that female parasites migrate at night to lay eggs near the anus, causing itching and discomfort. This nocturnal irritation may lead to restlessness during sleep, further contributing to bruxism in affected children [20]. This finding was inconsistent with those of previous studies [23, 45, 46] which reported that there was no significance association between bruxism and IPIs due to the complex multifactorial nature of bruxism.

Violent exposure is a significant indicator of bruxism due to its impact on psychological stress, emotional distress, and anxiety levels which are well-established risk factors for bruxism [47]. The observed IPI-bruxism association likely reflects a synergistic burden where parasitic exposure and the psychosocial stressors of resource-constrained settings—such as housing instability and chronic strain—amplify muscle hyperactivity. Consequently, IPIs may serve as a proxy for environmental deprivation, where these shared ‘upstream’ factors independently drive TMD and bruxism alongside the biological infection [48].

TMD was significantly associatd with positively infected group of children. This finding was consistent with previous studies [1, 24, 49–51], which could be attributed to repetitive excessive forces on the TMJ and masticatory muscles during bruxism. These combined effects may contribute to the development of pain, joint dysfunction, and other clinical features of TMD. TMD also increased with age, which is consistent with previous studies [52–54]. This may be driven by increased and cumulative biomechanical overload from bruxism due to recurrent infections. Another study finding was that TMD was significantly higher in female children, and this was also reported in previous studies [55, 56] due to greater pain sensitivity and reporting in females who were more exposed to recurrent IPIs.

It has also been reported that IPIs increased the risk of tooth wear significantly; these results are consistent with those of a previous study [57] which reported that tooth wear was higher in children with digestive disorders than children without. Tooth wear as possible sequel of bruxism was significantly higher in the positively infected group of children. This finding was in line with previous studies [24, 58–60]. The etiology behind tooth wear due to bruxism hinges on neuromuscular hyperactivity causing continuous tooth contact with excessive force beyond functional chewing. This leads to mechanical stresses that overwhelm the protective mechanisms of enamel, causing gradual structural loss. Higher maternal education has also been reported to be a protective factor against tooth wear. This may be due to educated mothers are more likely to instill regular and effective oral health practices, facilitating early dental visits, preventive treatments, and addressing risk factors promptly [61]. Other factors, including low sleep quality, adverse oral habits, stressful life events, and violent exposure, were not significantly associated with either condition.

This study has several strengths, including a relatively large, powered sample size which may provide higher statistical reliability than previous smaller-scale investigations in this field. A key point of originality in this research is the transition from subjective, binary parental reporting to the use of objective, validated clinical indices (Helkimo and Hugoson). While earlier research primarily focused on the habit of teeth grinding,

This study provides preliminary evidence on the association between intestinal parasitic infections and functional masticatory disorders in Egyptian children, highlighting an area that has been relatively underexplored. Furthermore, by evaluating both clinical signs and parental reports, the study provides a comprehensive assessment that aligns with modern multidimensional diagnostic frameworks.

The study has several limitations. One was that information was collected only from the accompanying parent, which may not necessarily be the parent who sleeps next to the child or who hears them during sleep. This concern is anticipated to have minimal impact on the accuracy of reporting. Additionally, more than one sample needs to be collected on three consecutive days to be more accurate in terms of the results. In addition, clinical examination of the TMJ was not easy in some young children and the most prominent symptoms were muscle pain and TMJ pain. In addition, owing to the cross-sectional design of the study, causality cannot be established and follow-up after taking anthelminthic drugs was much needed expecting recovery from bruxism. Furthermore, despite adjusting for maternal education, the complexity of socioeconomic status suggests that residual confounding from unmeasured environmental factors—which often coexist with IPIs—cannot be entirely ruled out as contributing factors. It should also be considered that tooth wear results from cumulative, multifactorial processes, highlighting the necessity for long-term future research. Additionally, the study population is limited to children attending a parasitological laboratory in Alexandria, Egypt, which may reduce the extent to which the findings are generalizable to other populations. Nevertheless, the study has several important findings.

The findings of this study have many implications for the diagnosis and management of TMD and tooth wear in children, particularly in regions where IPIs are prevalent. Clinicians should consider the possibility of parasitic infections in children presenting with TMD and tooth wear, especially when accompanied by other suggestive symptoms. Further research is needed to confirm these findings and elucidate the mechanisms underlying the association between IPIs and bruxism, TMD and tooth wear. Longitudinal studies could help establish the temporal relationship between parasitic infections and the development of bruxism, TMD and tooth wear. Additionally, studies investigating the impact of anthelminthic treatment on bruxism, TMD and tooth wear symptoms would provide further evidence for a causal link. Exploring the role of other potential contributing factors, such as diet, stress levels, and genetic predispositions, in the development of bruxism, TMD and tooth wear in children with and without IPIs would also be valuable.

## Conclusion

This study identifies a statistically significant association between IPIs and both bruxism, TMD and tooth wear among a group of Egyptian children. These findings suggest that IPIs may be relevant factors in the clinical presentation of these conditions, underscoring the importance of screening for parasites during pediatric dental evaluations. Further longitudinal research is warranted to validate these observations and clarify the underlying biological mechanisms.

## Supplementary Information

Supplementary Material 1.

## Data Availability

Data generated or analyzed from this study are available from the corresponding author upon reasonable request.

## Abbreviations

IPIs: intestinal parasitic infections
TMJ: temporomandibular joint
TMDs: temporomandibular disorders

## References

[1] De Oliveira Reis L, Ribeiro RA, Martins CC, Devito KL. Association between bruxism and temporomandibular disorders in children: A systematic review and meta-analysis. Int J Paediatr Dent. 2019;29:585–95. 10.1111/ipd.12496. 30888712 10.1111/ipd.12496

[2] Ohayon MM, Li KK, Guilleminault C. Risk factors for sleep bruxism in the general population. Chest. 2001;119:53–61. 10.1378/chest.119.1.53. 11157584 10.1378/chest.119.1.53

[3] Carlsson GE, Egermark I, Magnusson T. Predictors of bruxism, other oral parafunctions, and tooth wear over a 20-year follow-up period. J Orofac Pain. 2003;17:50–7. 12756931

[4] Caliskan S, Delikan E, A. O-K. Caliskan S, Delikan E, Ozcan-Kucuk A. Knowledge of Parents about Bruxism in their Children. Odovtos-Int J Dent Sc. 2020;22:123–32.

[5] Storari M, et al. Bruxism in children: What do we know? Narrative Review of the current evidence. Eur J Paediatr Dent. 2023;24:207–10. 10.23804/ejpd.2023.24.03.02. 37668461 10.23804/ejpd.2023.24.03.02

[6] Castroflorio T, et al. Risk factors related to sleep bruxism in children: A systematic literature review. Arch Oral Biol. 2015;60:1618–24. 10.1016/j.archoralbio.2015.08.014. 26351743 10.1016/j.archoralbio.2015.08.014

[7] Manfredini D, Ahlberg J, Lobbezoo F. Bruxism definition: Past, present, and future - What should a prosthodontist know? J Prosthet Dent. 2022;128:905–12. 10.1016/j.prosdent.2021.01.026. 33678438 10.1016/j.prosdent.2021.01.026

[8] Manfredini D, et al. Standardised Tool for the Assessment of Bruxism. J Oral Rehabil. 2024;51:29–58. 10.1111/joor.13411. 36597658 10.1111/joor.13411

[9] Manfredini A, et al. Sleep Bruxism Self-Report and Awake Bruxism: An Ecological Momentary Assessment. Oral Dis. 2025;31:3201–10. 10.1111/odi.15400. 40589372 10.1111/odi.15400 PMC12803566

[10] Okyay P, et al. Intestinal parasites prevalence and related factors in school children, a western city sample–Turkey. BMC Public Health. 2004;4:64. 10.1186/1471-2458-4-64. 15615592 10.1186/1471-2458-4-64 PMC544355

[11] World Health Organization (WHO). Soil-transmitted helminth infections. 2023. Available at: https://www.who.int/news-room/fact-sheets/detail/soil-transmitted-helminth-infections

[12] Tefera E, Mohammed J, Mitiku H. Intestinal helminthic infections among elementary students of Babile town, eastern Ethiopia. Pan Afr Med J. 2015;20:50. 10.11604/pamj.2015.20.50.5251. 26090008 10.11604/pamj.2015.20.50.5251 PMC4449975

[13] Yones DA, et al. Prevalence of gastrointestinal parasites and its predictors among rural Egyptian school children. J Egypt Soc Parasitol. 2019;49:619–30.

[14] Azzam A, Khaled H. Prevalence and risk factors of intestinal parasitic infections among preschool and school-aged children in Egypt: a systematic review and meta-analysis. BMC Public Health. 2025;25:2160. 10.1186/s12889-025-23325-8. 40495169 10.1186/s12889-025-23325-8 PMC12150492

[15] Elmonir W, et al. Prevalence of intestinal parasitic infections and their associated risk factors among preschool and school children in Egypt. PLoS ONE. 2021;16:e0258037. 10.1371/journal.pone.0258037. 34587187 10.1371/journal.pone.0258037 PMC8480785

[16] Al-Mohammed HI, et al. Prevalence of intestinal parasitic infections and its relationship with socio–demographics and hygienic habits among male primary schoolchildren in Al–Ahsa, Saudi Arabia. Asian Pac J Trop Med. 2010;3:906–12. 10.1016/S1995-7645(10)60218-0.

[17] Tehrani MH, et al. The Correlation between Intestinal Parasitic Infections and Bruxism among 3–6 Year-Old Children in Isfahan. Dent Res J (Isfahan). 2010;7:51–5. 22013457 PMC3177368

[18] Hajenorouzali Tehrani M, Sadri L, Mowlavi G. Intestinal Parasites and Bruxism in Children. Iran J Public Health. 2013;42:1199. 26060633 PMC4436553

[19] Shahbour SA, Abohamila N, EL-Bayoumi M. Prevalence of Sleep Bruxism and associated factors in Tanta Preschool Children. Alex Dent J. 2022;47:155–62. 10.21608/adjalexu.2022.72061.1187.

[20] EZIN DAM. Bruxism in children: Etiological causes, correlation with intestinal parasitic infestations, diagnosis and treatment criteria. World J Adv Res Rev. 2022;14:068–73.

[21] Ahmad R. Bruxism in children. J Pedod. 1986;10:105–26. 3458897

[22] Cazorla D, et al. Enterobius vermicularis infection in preschool and schoolchildren of six rural communities from a semiarid region of Venezuela: A clinical and epidemiological study. Helminthologia. 2006;43:81–5.

[23] Díaz-Serrano KV, et al. Is there an association between bruxism and intestinal parasitic infestation in children? J Dent Child (Chic). 2008;75:276–9. 19040814

[24] Bulanda S, et al. Sleep Bruxism in Children: Etiology, Diagnosis, and Treatment-A Literature Review. Int J Environ Res Public Health. 2021;18. 10.3390/ijerph18189544. 10.3390/ijerph18189544 PMC8471284 34574467

[25] Marks MB. Bruxism in allergic children. Am J Orthod. 1980;77:48–59. 10.1016/0002-9416(80)90223-7. 6928084 10.1016/0002-9416(80)90223-7

[26] Rosner B. Hypothesis Testing: Two-Sample Inference. In: Fundamentals of biostatistics. 7th ed. Boston: Brooks/Cole. Nelson Education. 2015;269–301.

[27] Universität, Düsseldorf. G*Power.2019. Retrieved from http://www.gpower.hhu.de/

[28] Clementino MA, et al. The prevalence of sleep bruxism and associated factors in children: a report by parents. Eur Arch Paediatr Dent. 2017;18:399–404. 10.1007/s40368-017-0312-x. 29075962 10.1007/s40368-017-0312-x

[29] Alves EG, Fagundes DM, Ferreira MC. Sleep bruxism in children and its association with clinical and sleep characteristics: cross-sectional study. RGO-Revista Gaúcha de Odontologia 2022;70.

[30] Emodi-Perlman A, et al. Bruxism, oral parafunctions, anamnestic and clinical findings of temporomandibular disorders in children. J Oral Rehabil. 2012;39:126–35. 10.1111/j.1365-2842.2011.02254.x. 21916926 10.1111/j.1365-2842.2011.02254.x

[31] Badahdah A, Le KT. Parenting Young Arab Children: Psychometric Properties of an Adapted Arabic Brief Version of the Alabama Parenting Questionnaire. Child Psychiatry Hum Dev. 2016;47:486–93. 10.1007/s10578-015-0581-8. 26323582 10.1007/s10578-015-0581-8

[32] Al-swaje N, Shammary A, shatha Al-khalifah SS, Shafshaks S. The prevalence of bruxism and dental wear in children in relation to smart devices and video games. Indo Am J P Sci. 2019;6:4560–5.

[33] Yağci İ, Taşdelen Y, Kivrak Y, Childhood, Trauma. Quality of Life, Sleep Quality, Anxiety and Depression Levels in People with Bruxism. Noro Psikiyatr Ars. 2020;57:131–5. 10.29399/npa.23617. 32550779 10.29399/npa.23617 PMC7285633

[34] Yusoff MSB. ABC of content validation and content validity index calculation. Educ Med J. 2019;11:49–54. 10.21315/eimj2019.11.2.6.

[35] Helkimo M. Studies on function and dysfunction of the masticatory system. II. Index for anamnestic and clinical dysfunction and occlusal state. Sven Tandlak Tidskr. 1974;67:101–21. 4524733

[36] Hugoson A, Bergendal T, Ekfeldt A, Helkimo M. Prevalence and severity of incisal and occlusal tooth wear in an adult Swedish population. Acta Odontol Scand. 1988;46:255–65. 10.3109/00016358809004775. 3264985 10.3109/00016358809004775

[37] Yazgan S, Çetinkaya Ü, Şahin İ. [The Investigation of Prevalence of Enterobius vermicularis (L.1758) in Primary School Age Children and Its Relation to Various Symptoms]. Turkiye Parazitol Derg. 2015;39:98–102. 10.5152/tpd.2015.3781. 26081881 10.5152/tpd.2015.3781

[38] Bertoncello C, et al. Sex-Biased Prevalence of Intestinal Parasitic Infections and Gender Inequality in Rural Nepal. Int J Infect Dis. 2021;109:148–54. 10.1016/j.ijid.2021.06.041. 34182133 10.1016/j.ijid.2021.06.041

[39] Rijal B, et al. Gender variations in the prevalence of parasitic infections and the level of awareness in adolescents in rural Nepal. Southeast Asian J Trop Med Public Health. 2001;32:575–80. 11944720

[40] Şentürk Ö, Güzel KGU, Tokgöz Y. Evaluation of relationship between Enterobius vermicularis infection and bruxism in children. Contemp Pediatr. 2022;3:61–72.

[41] da Silva Furtado Amaral C, et al. Bruxism in Childhood: A Scoping Review. J Calif Dent Assoc. 2024;52:2313775. 10.1080/19424396.2024.2313775.

[42] Almabadi ES, et al. Parental Sociodemographic Characteristics and Bruxism’s Risk Factors Among Children: Saudi Arabian Evaluation. Pediatr Health Med Ther. 2025;16:1–11. 10.2147/phmt.S471594. 10.2147/PHMT.S471594 PMC11720635 39802626

[43] Gonçalves JL, et al. Respiratory Problems and Different Manifestations of Bruxism in Children: A Retrospective Cross-Sectional Study. Pesquisa Brasileira em Odontopediatria e Clínica Integrada. 2025;25:e230214.

[44] Arbabi M, et al. Levels of Zinc, Copper, Magnesium Elements, and Vitamin B12, in Sera of Schoolchildren With Giardiasis and Entrobiosis in Kashan, Iran. Zahedan J Res Med Sci. 2015;17:e3659. 10.17795/zjrms-3659.

[45] Gilman RH, Marquis GS, Miranda E. Prevalence and symptoms of Enterobius vermicularis infections in a Peruvian shanty town. Trans R Soc Trop Med Hyg. 1991;85:761–4. 10.1016/0035-9203(91)90448-8. 1801349 10.1016/0035-9203(91)90448-8

[46] Montoya Castro V, Alvarez Chacón R, Pérez Romero BE, Chío MW. Prurito anal, nasal, bruxismo y sialorrea en niños con enterobiasis o ascariasis. Acta pediátr Méx. 1985:122–4.

[47] Girgla JK, et al. Exploring the Connection Between Domestic Violence and Masticatory Outcomes in the Pediatric Population: A Systematic Review. Cureus. 2023;15:e46764. 10.7759/cureus.46764. 37954731 10.7759/cureus.46764 PMC10632739

[48] Zahid Ullah Khan SKSKKKASAM. Incidence and Socioeconomic Impact of Intestinal Parasitic Infections among School Going Children of Private and Public Sectors Schools. Pak J Med Health Sci. 2022;16:1061. 10.53350/pjmhs221661061.

[49] Seraj B, et al. The Prevalence of Bruxism and Correlated Factors in Children Referred to Dental Schools of Tehran, Based on Parent’s Report. Iran J Pediatr. 2010;20:174–80. 23056700 PMC3446016

[50] Marpaung C, van Selms MKA, Lobbezoo F. Prevalence and risk indicators of pain-related temporomandibular disorders among Indonesian children and adolescents. Community Dent Oral Epidemiol. 2018;46:400–6. 10.1111/cdoe.12382. 29781511 10.1111/cdoe.12382

[51] Mortazavi N, Tabatabaei AH, Mohammadi M, Rajabi A. Is bruxism associated with temporomandibular joint disorders? A systematic review and meta-analysis. Evid Based Dent. 2023;24:144. 10.1038/s41432-023-00911-6. 37474733 10.1038/s41432-023-00911-6

[52] Minervini G, et al. Prevalence of temporomandibular disorders in children and adolescents evaluated with Diagnostic Criteria for Temporomandibular Disorders: A systematic review with meta-analysis. J Oral Rehabil. 2023;50:522–30. 10.1111/joor.13446. 36912441 10.1111/joor.13446

[53] Macrì M, Murmura G, Scarano A, Festa F. Prevalence of temporomandibular disorders and its association with malocclusion in children: A transversal study. Front Public Health. 2022;10:860833. 10.3389/fpubh.2022.860833. 36159244 10.3389/fpubh.2022.860833 PMC9500209

[54] Sena MF, et al. Prevalence of temporomandibular dysfunction in children and adolescents. Rev Paul Pediatr. 2013;31:538–45. 10.1590/s0103-05822013000400018. 24473961 10.1590/S0103-05822013000400018 PMC4183050

[55] List T, Wahlund K, Wenneberg B, Dworkin SF. TMD in children and adolescents: prevalence of pain, gender differences, and perceived treatment need. J Orofac Pain. 1999;13:9–20. 10425964

[56] Bagis B, et al. Gender difference in prevalence of signs and symptoms of temporomandibular joint disorders: a retrospective study on 243 consecutive patients. Int J Med Sci. 2012;9:539–44. 10.7150/ijms.4474. 22991492 10.7150/ijms.4474 PMC3444974

[57] Maharani DA, et al. Tooth wear among five-year-old children in Jakarta, Indonesia. BMC Oral Health. 2019;19:192. 10.1186/s12903-019-0883-5. 31429754 10.1186/s12903-019-0883-5 PMC6702728

[58] Gomes MC, et al. Evaluation of the association of bruxism, psychosocial and sociodemographic factors in preschoolers. Braz Oral Res. 2018;32:e009. 10.1590/1807-3107bor-2018.vol32.0009. 29412225 10.1590/1807-3107bor-2018.vol32.0009

[59] Dimitrova M, Rashkova M, Mitova N. Tooth wear in children-prevalence, clinical features and risk factors. J IMAB–Annual Proceeding Sci Papers. 2021;27:4020–4. 10.5272/jimab.2021274.4020.

[60] VÉLez AL, et al. Head posture and dental wear evaluation of bruxist children with primary teeth. J Oral Rehabil. 2007;34:663–70. 10.1111/j.1365-2842.2007.01742.x. 17716265 10.1111/j.1365-2842.2007.01742.x

[61] Duangthip D, et al. Erosive tooth wear among preschool children in Hong Kong. Int J Paediatr Dent. 2018. 10.1111/ipd.12457. 30565784 10.1111/ipd.12457

