# Supplementary material for: Intestinal parasitic infections and their association with bruxism, tooth wear, and temporomandibular disorders in children in rural Egypt: a cross-sectional study
Source: BMC Oral Health. 2026 Jun 4;26:1023. doi: 10.1186/s12903-026-08751-3 (PMC13262464; doi:10.1186/s12903-026-08751-3)
Supplement: Supplementary file 1 — Supplementary Material 1. [file 12903_2026_8751_MOESM1_ESM.docx]

**[Additional File 1]:**

| **Serial no.** |  |
| --- | --- |

**Date: ………**

**Place: ……….**

**Section 1**: **Demographic and personal data:**

- Child's name:
- Child’s age:
- Sex: Male/female:
- Parent's mobile number:
- Area of residency:

| Urban | Rural | Urban slums |
| --- | --- | --- |

- Mother’s education:

| Illiterate | Primary | Middle | Secondary | University | Higher |
| --- | --- | --- | --- | --- | --- |

- Father’s education:

| Illiterate | Primary | Middle | Secondary | University | Higher |
| --- | --- | --- | --- | --- | --- |

**Section 2: Interview**

**Bruxism and other oral habits:**

1. Does your child grind or clench his/ her teeth while asleep (at least once a week during the past 3 months).

| Yes | No | I Don't know |
| --- | --- | --- |

1. Does your child grind or clench during daytime or only during sleep?

| Yes | No | I Don't know |
| --- | --- | --- |

1. Does your child grind or clench on daily basis?

| Yes | No | I Don't know |
| --- | --- | --- |

1. Does your child suffer from any sleep disorders (e.g. obstructive sleep apnea syndrome)?

| Yes | No | I Don't know |
| --- | --- | --- |

1. Does your child often snore or see nightmares?

| Yes | No | I Don't know |
| --- | --- | --- |

1. Does your child bite down on hard objects (pens, pencils, etc.) or take apart toys ⁄games (e.g. Lego blocks) with the teeth?

| Yes | No | I Don't know |
| --- | --- | --- |

1. Does your child crush hard candies, ice, popsicles, etc. with the teeth or gum chewing?

| Yes | No | I Don't know |
| --- | --- | --- |

1. Does your child open bottles with the teeth?

| Yes | No | I Don't know |
| --- | --- | --- |

1. Does your child do‘Jaw-play’ (involuntary small mandibular movements without tooth contact)?

| Yes | No | I Don't know |
| --- | --- | --- |

**Stressful life events and violent exposure:**

1. Birth of a sibling

| Yes | No |
| --- | --- |

1. Change of address

| Yes | No |
| --- | --- |

1. Divorce of the parents

| Yes | No |
| --- | --- |

1. Death of a family member

| Yes | No |
| --- | --- |

1. Others

| Yes | No |
| --- | --- |

f. Is your child physically or emotionally abused in any way (hittig your child with an abject/ spanking your child with your hand/ slapping your child/ threatening your child if he/ she has done something wrong)?

| Yes | No | I Don't know |
| --- | --- | --- |

g.Is your child interested in violent video games?

| Yes | No | I Don't know |
| --- | --- | --- |
